# SLC2A3 promotes tumor progression through lactic acid-promoted TGF-β signaling pathway in oral squamous cell carcinoma

**DOI:** 10.1371/journal.pone.0301724

**Published:** 2024-04-16

**Authors:** Wei Jiang, Sheng Xu, Ping Li

**Affiliations:** 1 Guangxi Key Laboratory of Early Prevention and Treatment for Regional High Frequency Tumor, Guangxi Medical University, Nanning, Guangxi Zhuang Autonomous Region, China; 2 College of Stomatology, Guangxi Medical University, Nanning, Guangxi Zhuang Autonomous Region, China; 3 Department of Dental Laboratory, Guangxi Medical University College of Stomatology, Nanning, Guangxi Zhuang Autonomous Region, China; 4 Department of Pathology, Guangxi Medical University College of Stomatology, Nanning, Guangxi Zhuang Autonomous Region, China; Università degli Studi della Campania, ITALY

## Abstract

**Backgrounds:**

Oral squamous cell carcinoma is a malignant tumor of the head and neck, and its molecular mechanism remains to be explored.

**Methods:**

By analyzing the OSCC data from the TCGA database, we found that SLC2A3 is highly expressed in OSCC patients. The expression level of SLC2A3 was verified by RT-PCR and western blotting in OSCC cell lines. The function of SLC2A3 in OSCC cell lines and Lactic acid in SLC2A3-knockdown OSCC cells were detected by colony formation, CCK8, transwell, and wound healing assays. The effect of SLC2A3 on tumor growth and metastasis was tested in vivo. GSEA and Western blot were used to analyze and validate tumor phenotypes and signaling pathway molecules.

**Results:**

We analyzed OSCC datasets in the TCGA database and found that SLC2A3 had abnormally high expression and was associated with poor prognosis. We also found that oral squamous cell carcinoma cells had increased proliferation, migration, invasion, EMT phenotype, and glycolysis due to SLC2A3 overexpression. Conversely, SLC2A3 knockdown had the opposite effect. Our in vivo experiments confirmed that SLC2A3 overexpression promoted tumor growth and metastasis while knockdown inhibited it. We also observed that high SLC2A3 expression led to EMT and the activation of the TGF-β signaling pathway, while knockdown inhibited it. Interestingly, exogenous lactic acid restored the EMT, proliferation, migration, and invasion abilities of oral cancer cells inhibited by knocking down SLC2A3.

**Conclusions:**

Our study reveals that SLC2A3 expression was up-regulated in OSCC. SLC2A3 activates the TGF-β signaling pathway through lactic acid generated from glycolysis, thus regulating the biological behavior of OSCC.

## Introduction

Oral cancer ranks as the eighth most common cancer globally, with more than 350,000 new cases and 170,000 fatalities reported in 2018 [1, 2]. Oral squamous cell carcinoma (OSCC), constituting over 95% of all oral cancer cases, has seen an increasing incidence, especially among younger populations [3, 4]. This trend underscores the urgent need for research into the mechanisms of OSCC’s onset and progression to advance prevention and treatment strategies.

*SLC2A3* plays a crucial role in oral cancer development by facilitating glucose transport across cell membranes, thus supporting the enhanced glycolytic activity necessary for rapid tumor cell proliferation. This aberrant metabolic behavior, characterized by heightened glucose uptake, is a hallmark of malignancy [5]. Prior research has primarily concentrated on clinical prognosis and has pointed out that overexpression of *SLC2A3* is prominently associated with worse prognosis, such as increased depth of invasion, larger tumor size, advanced pathological stage, recurrence, and vascular embolization [6]. Moreover, the expression levels of *SLC2A3* have been identified as a significant prognostic marker for disease-free, relapse-free, and overall survival, with higher expression levels correlating with poorer disease-free survival and more aggressive tumor characteristics [7]. Despite these insights, there remains a substantial gap in our understanding of the specific molecular mechanisms influenced by *SLC2A3* and its associated molecules in OSCC progression and prognosis.

Lactic acid(LA), a byproduct of glycolysis mediated by *SLC2A3*, is a critical intermediary in this process. Elevated lactic acid levels in tumor biopsy samples have been consistently linked to greater invasive capacity [8–11]. Recent studies highlight lactic acid’s role in promoting cancer progression through intricate molecular signaling pathways, crucial for tumorigenesis [12]. In oral cancer, lactic acid contributes to significant molecular alterations, including the loss of E-cadherin and the increase of mesenchymal markers like N-cadherin and Vimentin, which are essential for the epithelial-mesenchymal transition (EMT) [13]. Lactic acid also accelerates basement membrane remodeling by facilitating the degradation of key structural components [14, 15]. Nevertheless, the specific role of lactic acid as a downstream product of *SLC2A3* in glycolysis and its impact on OSCC remain largely unexplored.

This study presents novel insights into the significant role of *SLC2A3* signaling and the consequential regulation by LA in OSCC progression. Our findings aim to elucidate the important role of *SLC2A3* signaling and reveal the regulating mechanism of LA mediated in the progression of OSCC, offering potential pathways for early prediction and novel therapeutic strategies in OSCC management.

## Materials and methods

### Bioinformatics analysis

Gene expression matrix and clinical information of 340 oral squamous cell carcinoma patients were downloaded from the HNSC datasets of the TCGA database(https://portal.gdc.cancer.gov/). Age, prognostic stage, TNM stage, tumor grade, survival status, and lymphovascular and perineural invasion were contained in clinical data. Cases with missing information were excluded. All datasets in the present study were downloaded from a public database, allowing researchers to download and analyze public datasets for scientific purposes.

Survival and ROC analyses were conducted with data from aclbi(www.aclbi.com), an online tool based on the TCGA database.

### Clinical samples

Twelve pairs of OSCC tissues and adjacent non-tumor tissues were collected over a six-month period starting from March 14, 2023. The collected samples were anonymized to ensure participant confidentiality. Each biopsy was fixed in 10% paraformaldehyde, embedded in paraffin, and subsequently diagnosed by an expert pathologist based on the World Health Organization (WHO) classification criteria.

The study was approved by the Research Ethics Committee of First Affiliated Hospital of Guangxi Medical University (No.2023-K064-01). Written informed consent was obtained from all patients whose information was reasonably protected and not identified. All methods were performed in accordance with the relevant guidelines and regulations. The study was performed in accordance with the Declaration of Helsinki.

### Cell culture, SLC2A3 knockdown and overexpression

Oral squamous cell carcinoma (OSCC) cell lines (SCC9, SAS, and CAL27) along with normal human oral squamous epithelial cells (NOK) were cultured in DMEM media (Invitrogen, USA) supplemented with 10% fetal bovine serum, 100 U/mL of penicillin, and 100 mg/mL of streptomycin. These cells were incubated at 37°C in a humidified atmosphere containing 5% CO2 until they reached 70% confluence. At this point, siRNA against *SLC2A3* (si-*SLC2A3*, sequence CCUGAGAAGAUCAUAAAGGAATT), pcDNA-*SLC2A3* vector (sequence CCUGAGAAGAUCAUAAAGGAATT), and respective negative control plasmids were transfected into the cells using Lipofectamine 2000 (Invitrogen, USA). For comparison, non-transfected wild-type SCC9 or SAS cells served as blank controls. For in vivo studies, GFP-tagged sh*SLC2A3*, pcDNA-*SLC2A3*, and control plasmids were packaged into lentiviruses for infection of SCC9 or SAS cell lines.

### Quantitative real-time PCR

Total RNA from OSCC cells or tissues was isolated using TRIzol reagent (Invitrogen, USA), followed by cDNA synthesis according to the TransScript First-Strand cDNA Synthesis SuperMix (TransGen, China) protocol. Amplification of cDNA was performed using specific primers for *SLC2A3* (Forward: 5′-CCUGAGAAGAUCAUAAAGGAATT-3′, Reverse: 5’-UUCCUUUAUGAUCUUCUCAGGTT-3’) and GAPDH (Forward: 5’-TCAAGGCTGAGAACGGGAAG-3’, Reverse: 5’‐TGGACTCCACGACGTACTCA-3’). The expression levels of target genes were quantified using the 2^-ΔΔCT^ method based on Ct values.

### Immunohistochemical staining

Deparaffinized and rehydrated tissue sections (3-μm thick) were treated with 3% hydrogen peroxide for one hour to block endogenous peroxidase activity, followed by antigen retrieval. The sections were incubated overnight at 4°C with antibodies against *SLC2A3* (1:400, Santa Cruz, USA), *E-cadherin* (1:200, CST, USA), *N-cadherin* (1:200, CST, China), *Vimentin* (1:100, CST, China), and *Ki67* (1:200, ZSGB, China). After exposure to a secondary antibody (ZSGB, China) for one hour at room temperature, a 3,3′-diaminobenzidine (DAB) reagent was applied for visualization, followed by hematoxylin counterstaining. Images were acquired under an Olympus microscope, and two independent pathologists blindly assessed the staining results.

### Western blot

Protein isolation was carried out using RIPA buffer (Beyotime Biotechnology, China) supplemented with a protease inhibitor cocktail (Fabio Science, China). Proteins, each weighing 120mg, were electrophoresed on a 4–12% SDS-PAGE gel, with 5mg Protein ladder(26619/26620, Thermo Fisher Scientific, USA), and subsequently transferred onto a PVDF Membrane (Thermo Fisher Scientific, USA). The membrane was cut into fragments according to the kDa of the target protein. Post-blocking the membranes with 5% BSA, these fragments were applied to different primary antibodies simultaneously according to their kDa. Afterward, secondary antibodies (anti-rabbit/mouse, diluted at 1:10000, Licor, USA) were used for 2 hours at room temperature. GAPDH served as an internal control. These fragments that comes from the same and complete membrane were finally put together according to the order of molecular weight marker during imaging(Bio-rad ChemiDoc Touch, Bio-rad, USA). Blot images were loaded into ImageJ, converted to 8-bit type, and quantified using the gel function within the Analyze module. The quantization of blot was then obtained using the Wand tool, and relative protein expression was calculated by the formula: relative protein expression = Objective blot area value/corresponding GAPDH area value.

### Colony formation assay

In 6-well plates, 300 OSCC cells were placed per well. The culture medium was renewed every three days, and a two-week incubation at 37°C followed. The cells were then rinsed thrice with PBS and set with 4% paraformaldehyde for half an hour. Following this, Giemsa’s solution was used to stain the cells for another 30 minutes, after which colony formations were photographed and loaded into ImageJ software. Binary images were created by thresholding the images to remove background noise. The counts and size of colony formations were analyzed and calculated using the analyze particle tool in the Analyze module of ImageJ (size range: 20-infinity pixels), resulting in counts and average area.

### Cell proliferation assay

A CCK‐8 assay (Cell counting kit‐8, Dojindo, Japan) was used to assess the proliferation of OSCC cells and half maximal inhibitory concentration of lactic acid. In 96‐well plates, 1000 cells per well were cultured and incubated at 37°C. Afterward, each well received 100 μL of CCK‐8 buffer, followed by a 2-hour incubation at 37°C. The optical density (OD) was measured at 450 nm using a microplate reader (BIO‐TEK, USA).

### Wound healing assay

Cells were seeded at a density of 3×10^5^ per well into 12-well plates using DMEM media minus FBS overnight. Monolayer cells were scratched using an ibidi culture insert (ibidi, Germany). Images were taken under an inverted phase microscope (TS100, Nikon, Japan) at the start (0 h) and after 8 hours. Image J ver.1.51k (NIH, USA) software was used to analyze the migration rate.

### Transwell assay

Cells (4 × 10^6^ per well) were seeded in the upper chamber of 24-well Bio-Coat Invasion Chambers (8μm pores, BD, USA) coated with Matrigel. The lower chamber was filled with DMEM medium with 10% FBS. Non-invading cells were removed by using a cotton-tipped swab at 48 h. Migratory and invasive cells on the lower membrane surface were fixed with 1% paraformaldehyde, stained with 0.5% crystal violet, and photographed.

### Morphological analysis of epithelial-mesenchymal transition

In order to describe morphological changes in SCC9 and SAS cells, we did area and aspect ratio measurements using Fiji ImageJ software. The area measurements were performed on the binary images using the ‘Wand (tracing) tool’ in Fiji ImageJ. A hundred cells were randomly captured and calculated the average area in each group. The aspect ratio of cells was calculated based on their major and minor axes. The major axis refers to the longest axis inside the cell, while the minor axis is the perpendicular axis to the major axis and is located in the middle of the cell.To calculate the aspect ratio, the length of the major axis was divided by the length of the minor axis for each of the 10 randomly selected cells.

### In vivo tumorigenesis and metastasis

The animal study was approved by the Animal Care & Welfare Committee of Guangxi Medical University (No. 202302002), adhering to established guidelines. Thirty-six male BALb/c nude mice, five weeks old, obtained from the Experimental Animal Center of Guangxi Medical University (Nanning, China), received subcutaneous injections in the right axillary region with 5×10^7 SCC9 or SAS cells. These cells were pre-treated with GFP-tagged sh*SLC2A3* (sh-*SLC2A3*), pcDNA-*SLC2A3* (*SLC2A3*), or negative control (NC) lentiviruses. Tumor measurements commenced on the eighth-day post-injection, with subsequent records every four days using the formula: volume (mm^3) = 0.5×length×width^2. On the forty-eighth day, all mice were euthanized, their tumors harvested and weighed.

For intravenous injections, eighteen male BALb/c nude mice were administered SCC9 or SAS cells (4x10^7 cells/200μL) via the tail vein. These cells were labeled with fluorescent sh-*SLC2A3*, *SLC2A3*, or NC. Organ metastasis was monitored weekly using in vivo imaging (BLT Aniview, China), with the timing of occurrence noted in weeks.

Mice receiving tumor cell injections were euthanized after 48 days, whereas those receiving tail vein injections were observed for up to five months. Experiments were terminated early if mice exhibited severe symptoms or pain to minimize suffering. Euthanasia criteria included:1)A tumor diameter exceeding 15 mm. 2)Tumor burden surpassing 15% of body weight. 3)A body weight loss of 10%.

All animals were euthanized via cervical dislocation following proper anesthesia.

### Gene set enrichment analysis

The OSCC dataset from TCGA, comprising 340 samples, was subjected to gene set enrichment analysis using GSEA software version 4.0.3. The specific gene set "h.all.v6.1.symbols.gmt," which encapsulates a well-defined biological state or process, was retrieved from the GSEA database. The mRNA expression levels of SLC2A3 were categorized into low and high groups, and the analysis included 1000 permutations of the gene set. Results were considered statistically significant when the false discovery rate (FDR) was below 0.25 and the p-value was less than 0.05.

### Glycolysis assay

The concentrations of glycolytic products, including lactic acid, intracellular glucose, and ATP, were determined using test kits (Jiancheng, China) as per the manufacturer’s guidelines.

### Statistical analysis and data repository

All statistical analyses were performed using SPSS version 22.0 (SPSS, USA). The *Student’s t-test* and *one-way ANNOVA* were employed to evaluate statistical significance, with a *p*-value less than 0.05 denoting significance. Data are presented as means of three independent repeats ± standard deviations (SD). Significance levels were denoted as: * for p < 0.05, ** for p < 0.01, *** for p < 0.001, and ns for no significance. All the relevant raw images and data have been uploaded to doi: 10.6084/m9.figshare.25259614 in accordance with *the minimal data set by PLOS ONE*.

## Results

### *SLC2A3* is abnormally highly expression in OSCC and predicts worse clinical outcomes

**Fig 1:**
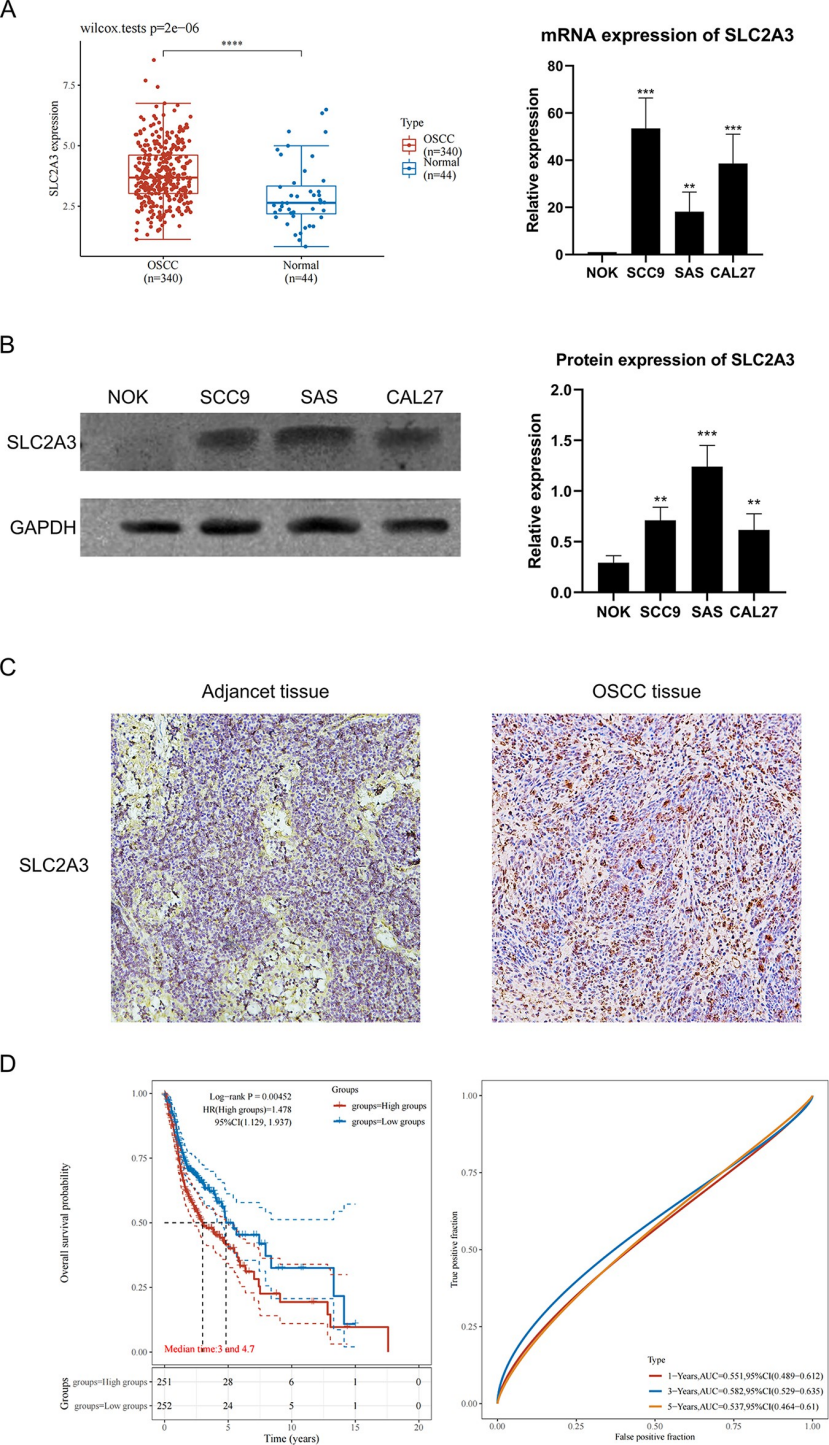
*SLC2A3* is abnormally highly expressed in OSCC and predicts worse clinical outcomes. (A) At the RNA level, the expression of SLC3A3 was demonstrated in both microarrays (TCGA data source) and cell lines, respectively. Normal tissue and NOK cells served as controls. (B) At the protein level, the expression of SLC2A3 in OSCC cell lines and NOK cells is illustrated in bar charts. Data show means of three independent reports, and error bars indicate standard deviation in (A) and (B). (C) Representative immunohistochemical images of SLC2A3 are in the Adjacent and the OSCC issue, respectively(×400). (D) The clinical significance of SLC2A3 is reflected by survival analysis (left) and ROC (right). Kaplan Meier survival analysis of the SLC2A3 high and low groups of samples from the TCGA dataset, comparison among different groups was made by log-rank test HR (95% Cl), the median survival time for different groups. The ROC curve of the gene at 1,3,5 years The higher values of AUC responding to higher predictive power.

This study first assessed the expression of *SLC2A3* in oral cancer tissues and cells. Analysis of OSCC patient data from the HNSC database in TCGA (Fig 1A) revealed that *SLC2A3* was markedly overexpressed in OSCC tissues. Furthermore, at the cellular level, oral squamous carcinoma cell lines (SCC9, SAS, CAL27) exhibited higher *SLC2A3* expression compared to normal human oral squamous epithelial cells (NOK). These findings suggest that *SLC2A3* is abnormally upregulated at both RNA and protein levels in oral cancer.

Protein expression analyses (Fig 1B and 1C) corroborated that *SLC2A3* was significantly overexpressed in both OSCC cell lines and tissues. Integrating these findings with clinical data from TCGA (Fig 1D, Table 1), it was observed that patients with elevated *SLC2A3* expression had a reduced overall survival rate and higher expression levels were noted in patients with advanced T and N stages, pathologic grade, clinical grade, and tumor grade. These results indicate that high *SLC2A3* expression may be an influential factor in tumor progression and could be a critical diagnostic marker for oral cancer, associated with a poor prognosis.

**Table 1:** The correlation between *SLC2A3* mRNA expression and clinicopathological features of OSCC patients based on the TCGA database.

Variable	n(%)	mRNA expression of SLC2A3		
		M	SD	*P*-value
Survival status				
Alive	187(55%)	4.8130	5.7142	0.011*
Dead	153(45%)	7.0057	9.84063	
Pathological stage				
I-II	76(22.3%)	5.1445	3.76143	0.031*
III-IV	233(68.5%)	6.1006	4.48372	
Clinical stage				
I-II	94(27.6%)	4.1947	3.30362	0.015*
III-IV	237(69.7%)	6.9204	11.04351	
T stage				
T0-T1	128(37.6%)	4.9130	5.98648	0.043*
T2-T3	201(59.1%)	6.7957	11.07501	
N stage				
N0-N1	232(68.1%)	5.1434	7.76892	0.020*
N2-N3	94(27.6%)	8.1449	12.27866	
M stage				
M0	321(94.4%)	6.1651	9.67856	0.342
M1	2(0.5%)	4.7782	5.27517	
Grade				
I-II	257(75.4%)	5.5385	7.96067	0.049*
III-IV	74(21.6%)	7.9354	13.23012	
Lymphovascular invasion				
Yes	75(22.0%)	6.6510367	6.42585167	0.369
No	167(49.7%)	5.4206673	6.36179486	
Perineural invasion				
Yes	137(40.2%)	7.025724544	6.546865846	0.241
No	120(35.2%)	5.700778936	11.17603827	

### *SLC2A3* promote the growth, invasion and migration of OSCC cells

**Fig 2:**
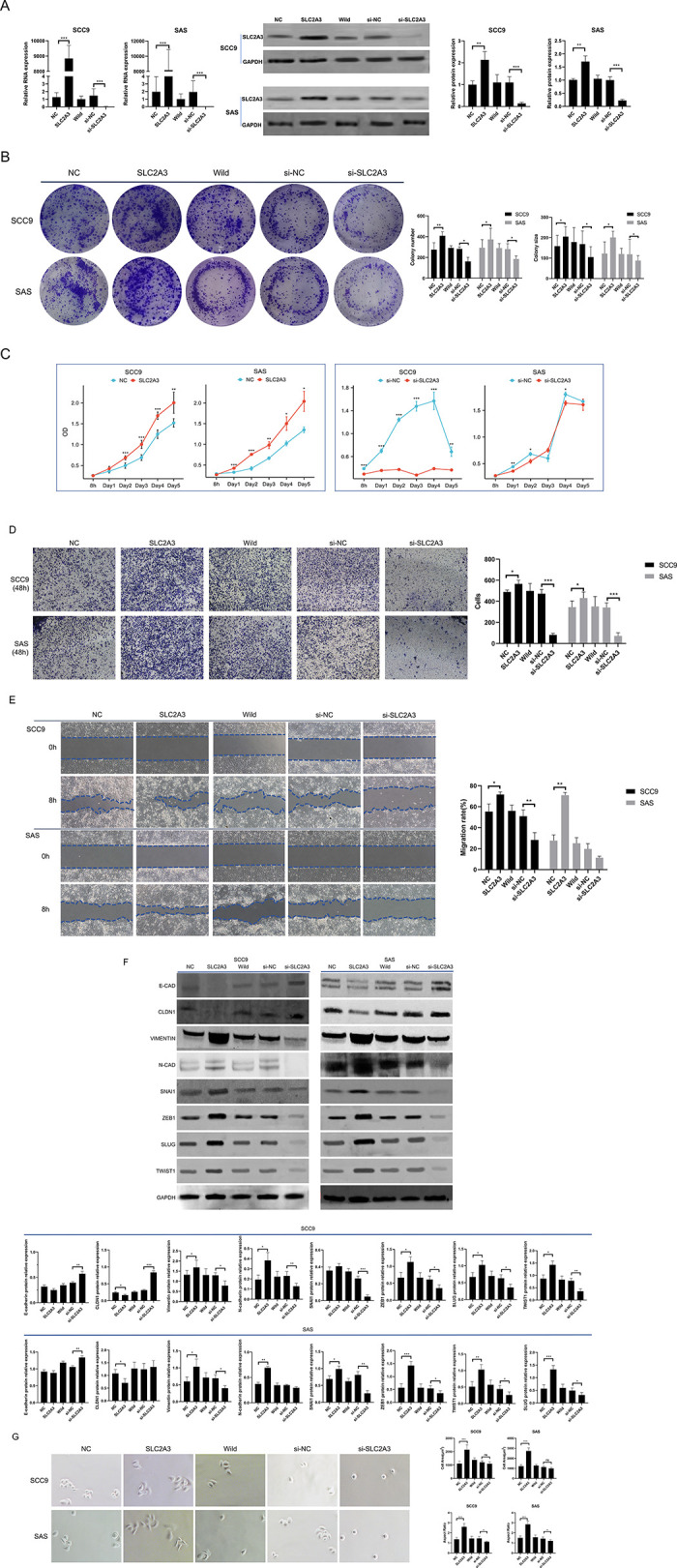
*SLC2A3* promote the growth, invasion and migration of OSCC cells. (A) SLC2A3 was successfully knocked down and overexpressed at the RNA and protein levels in SCC9 and SAS cells. (B-E) Cell growth was detected by colony formation and cell count. And the invasion, migration were detected by transwell and wound healing expriments, respectively. (F,G) These EMT-related protein levels were detected by western blot, while morphological changes(×1000) were analyzed by cell area and aspect ratio. In these analyses, the overexpression groups SLC2A3 and si-SLC2A3 were compared with their negative controls, respectively. Data are presented as means of three independent repeats ± standard deviations (SD).

This study investigated the functional impact of *SLC2A3* in OSCC by regulating its expression levels in SCC9 and SAS cells. The expression of *SLC2A3* was verified by PCR and Western blot at RNA and protein levels (Fig 2A). We found that cell viability and proliferation were significantly enhanced by *SLC2A3* overexpression and significantly diminished by knockdown (Fig 2B and 2C). These results suggest that relatively high expression of *SLC2A3* enhances OSCC cell proliferation and activity.

Additionally, the capacity of cells to invade and migrate, crucial for oral cancer progression, was assessed through Transwell and Migration assays. Enhanced invasive and migratory rates were associated with *SLC2A3* overexpression, whereas its knockdown yielded inverse outcomes (Fig 2D and 2E). Western Blot analysis revealed a decrease in epithelial markers (e.g., *E-cadherin and CLDN1*) and an increase in mesenchymal markers (e.g., *N-cadherin*, *Vimentin*, *SNAI1*, *ZEB1*, *TWIST*, *and SLUG*) upon *SLC2A3* overexpression. The opposite pattern was observed when *SLC2A3* expression was knockdown (Fig 2F). Morphological changes indicative of epithelial-to-mesenchymal transition were also altered by modulating *SLC2A3* levels. Cells overexpressing *SLC2A3* exhibited increased cell area and aspect ratio, whereas *SLC2A3* knockdown resulted in smaller, rounder cells (Fig 2G). These findings correlate *SLC2A3* with OSCC cell proliferation, invasion capability, and EMT phenotype.

### *SLC2A3* promotes glycolysis in OSCC

**Fig 3:**
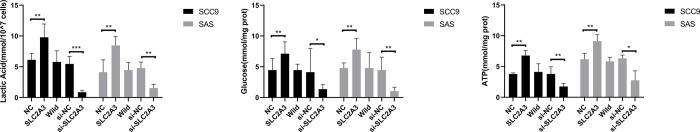
*SLC2A3* promotes glycolysis in OSCC. The impact of overexpressed and knocked-down *SLC2A3* on the intracellular levels of Lactic Acid (A), Glucose (B), and ATP (C). The overexpression groups *SLC2A3* and si-*SLC2A3* were compared with their negative controls, respectively.

Examination of intracellular lactic acid, glucose, and ATP levels revealed that *SLC2A3* overexpression led to amplified production of glycolytic by-products and increased glucose consumption, while the opposite effects were noted upon *SLC2A3* knockdown (Fig 3). This suggests that *SLC2A3* plays a pivotal role in regulating glucose metabolism, thereby fueling the proliferation of tumor cells.

### *SLC2A3* enhance tumor growth and metastasis in vivo

**Fig 4:**
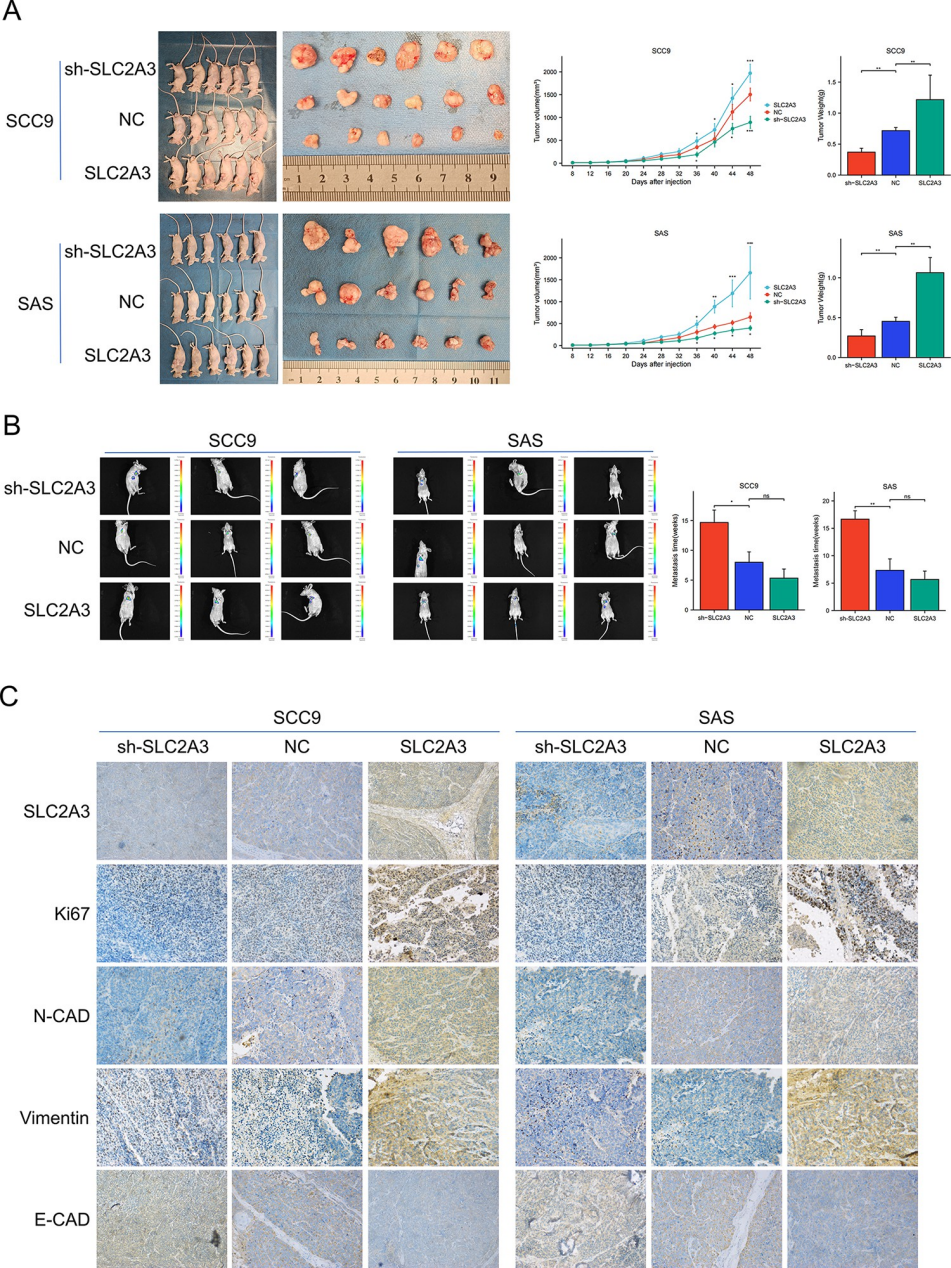
*SLC2A3* enhances tumor growth and metastasis in vivo. (A)In BALB/c nude mice, SCC9 and SAS cells with overexpressing, knocking down *SLC2A3* and negative control(NC) were, respectively, injected subcutaneously to analyze the tumor volume and mass. The tumor volume is calculated using the formula: volume (mm^3) = 0.5 × length × width^2. At the same time point, the tumor volumes of the overexpression and the knockdown group were compared with the NC group, respectively. Similarly, the same comparsions were conducted in tumor weight. (B) These cells were also rejected into the tail vein to observe the occurrence of tumor metastasis (in weeks). Both overexpression and knockdown group were compared with NC group, respectively.(C) The proteins expression of SLC2A3, Ki67, N-CAD, Vimentin, and E-CAD were detected by immunohistochemistry (× 400).

SCC9 and SAS cells, engineered to overexpress or knock down *SLC2A3*, were subcutaneously injected into BALb/c nude mice, and subsequent tumor volume and weight measurements were recorded. The findings demonstrated that tumor volume and weight significantly increased in the mice injected with *SLC2A3*-overexpressing cells (Fig 4A). Additionally, the tail vein metastasis assay revealed a delayed onset of metastasis in the *SLC2A3* knockdown group (Fig 4B). Immunohistochemical analysis further indicated that *SLC2A3* knockdown reduced the expression of Ki67, Vimentin, and N-cadherin, while upregulating E-cadherin expression (Fig 4C). These observations imply that downregulating *SLC2A3* expression curtails tumor growth and metastasis.

### *SLC2A3* regulates malignant behavior of OSCC cells through the TGF-β pathway

**Fig 5:**
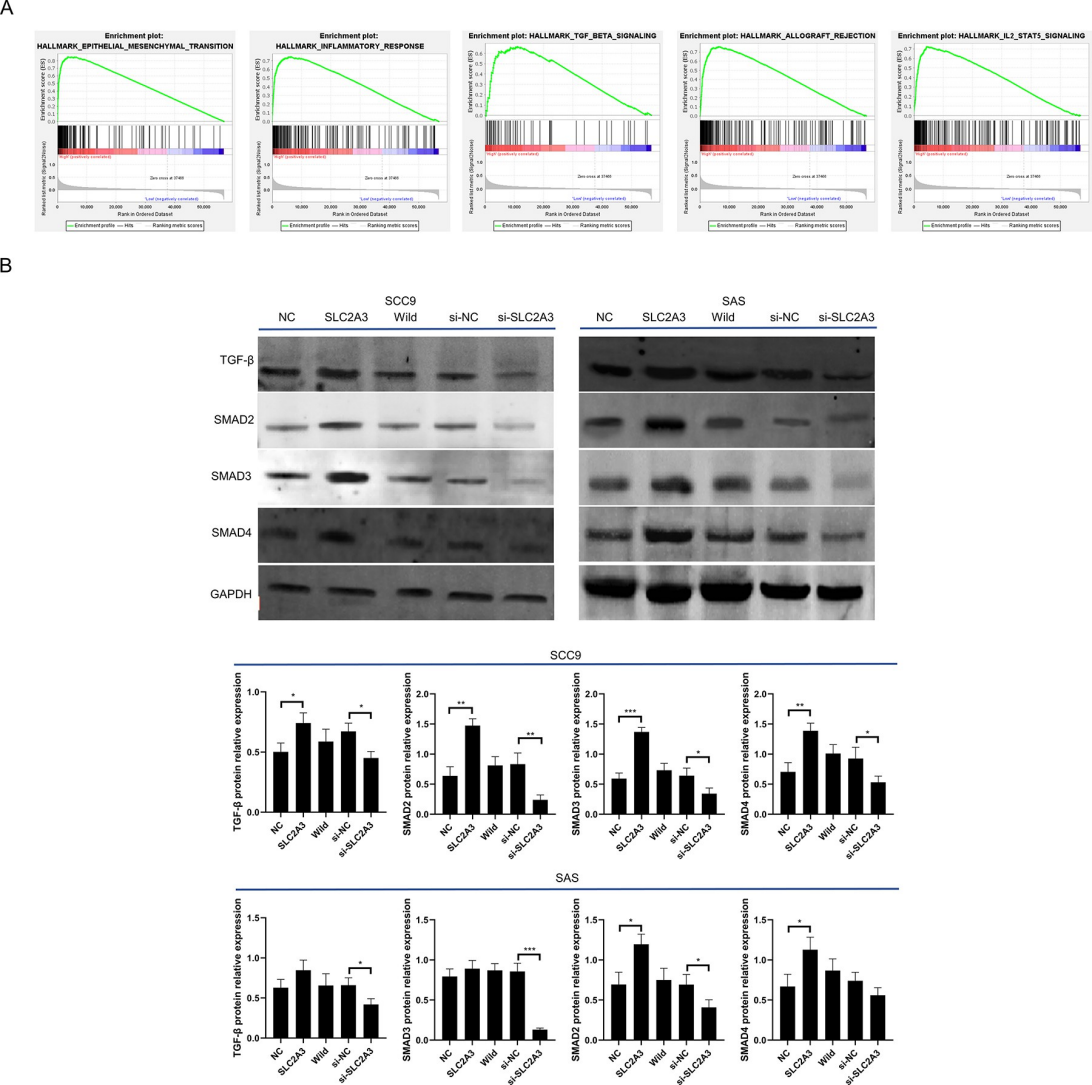
*SLC2A3* regulates malignant behavior of OSCC cells through the TGF-β pathway. (A) Results of GSEA analysis of highly expressed *SLC2A3*, items with p < 0.05 are ranked from highest to lowest enrichment score. (B) The major signaling pathway among the screened items was validated by western blot. The related proteins of TGF-β signaling pathway including TGF-β, SMAD2, 3, 4 were illustrated in blots along with GAPDH as internal control. The overexpression groups *SLC2A3* and knockdown group si-*SLC2A3* were compared with their negative controls, respectively.

To investigate the mechanism of the role of *SLC2A3* on malignant biological behaviors, 340 oral cancer patients from TCGA were categorized into high and low *SLC2A3* expression cohorts based on median expression levels. Gene Set Enrichment Analysis (GSEA) identified five pathways significantly associated with high *SLC2A3* expression, listed in descending order by enrichment score (ES): epithelial-mesenchymal transition (EMT), inflammatory response, TGF-β signaling, graft rejection, and the IL2 STAT5 signaling pathway(Fig 5A). Among the signaling pathways, the primary enrichment was in TGF-β. Validation by Western Blot further revealed that *SLC2A3* overexpression activates the TGF-β signaling pathway, whereas *SLC2A3* knockdown inhibits it (Fig 5B). These findings suggest that *SLC2A3* expression influences the malignant behavior of OSCC cell through modulation of the TGF-β pathway

### Lactic acid is a mediator of TGF-β regulation by *SLC2A3*

**Fig 6:**
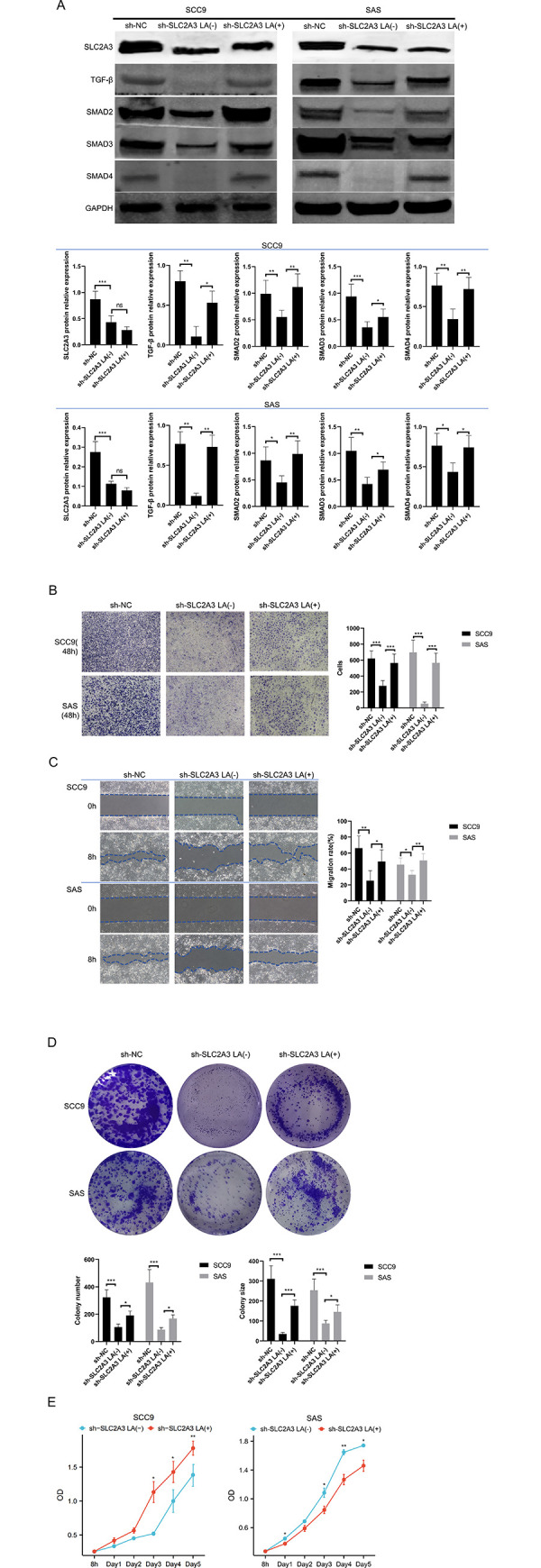
Lacitc acid is a mediator of TGF-β regulation by *SLC2A3*. (A) The expression of *SLC2A3* and TGF-β signaling pathway in *SLC2A3*-knockdown cells was detected by western blot after the lactic acid was added. (B,C) Cell invasion and migration were detected by transwell and would healing experiment, respectively. (D,E) The growth was detected by colony formation, and cell count. The comparsion of two pairs(sh-NC and sh-*SLC2A3* LA(-); sh-*SLC2A3* LA(-) and LA(+)) were respectively performed in these analysis.

To determine if lactic acid serves as a facilitator for the activation of TGF-β by *SLC2A3*, OSCC cells with knocked-down *SLC2A3* were treated with exogenous lactic acid at concentrations derived from IC50 values (S1 Fig). Western blot experiments showed that the addition of exogenous lactic acid did not affect *SLC2A3* expression but activated the TGF-β/SMAD2/3/4 signaling pathway, indicating that lactic acid act as a re-activator and mediator for TGF-β signaling pathway.(Fig 6A). Further investigation into the effects of lactic acid on the biological behavior of *SLC2A3*-knockdown cells involved conducting Transwell and Migration assays to assess invasive and migratory capabilities, as well as plate cloning and CCK8 assays to evaluate proliferation. Results indicated that although *SLC2A3* knockdown reduced migration, invasion, and proliferation, the exogenous lactic acid facilitated a recovery in these cellular functions (Fig 6B–6E).

## Discussion

Our study identifies *SLC2A3* as an oncogene significantly upregulated in OSCC, correlating with a worse prognosis. Consistently reported in the literature, *SLC2A3* shows overexpression in the basal and suprabasal layer cells of OSCC, demonstrating increased glucose uptake [16]. Also, bioinformatics analyses by Chu predict that upregulated *SLC2A3* may link to advanced clinical stages and lymph node metastasis [17]. Fernanda’s IHC analysis confirms high *SLC2A3* expression is associated with advanced tumor stages, vascular invasion, and decreased disease-free survival [16]. However, the association of increased expression of a glucose transporter with cancer also could be explained by the Warburg effect, which most carcinomas were underly. In this case, increased expression of *SLC2A3* may be a byproduct of tumorigenesis without being causally related to prognosis. In our results, the up-and-down-regulatory functional experiments revealed that *SLC2A3* could comprehensively underpin the cancer-promoting aspects in both vivo and vitro, providing a preliminary basis for its cancer-facilitating properties.

Despite these findings, a gap remains in understanding the mechanisms linking abnormal expression with its effects. Lactic acid, a downstream molecule of *SLC2A3* in glycolysis, emerges as a critical factor. Our data show that overexpression of *SLC2A3* significantly increases lactic acid concentration, which in turn facilitates malignant behaviors. Notably, elevated lactic acid levels in tumors have been associated with enhanced tumor growth through various mechanisms [12]. Immunologically, lactic acid diminishes the anticancer capabilities of NK and NKT cells and cytokines, thereby fostering tumor proliferation [18]. In terms of invasion, at adequate concentrations, lactic acid binds to GPR81 and enhances its expression via extracellular signaling and transcriptional activation through the 3stail3/STAT3 pathways, promoting tumor angiogenesis (VEGF) and immune evasion (PD-1) [19–24]. Furthermore, lactic acid acts as a signaling molecule, upregulating cathepsin B, MMP-9, and MMP-2 expression, which contribute to the degradation of collagen IV and VII and glycoproteins, thus facilitating basement membrane remodeling [25–27]. It also promotes epithelial-mesenchymal transition (EMT) through the TGF-β/SMAD, Wnt/β-catenin, IL-6/STAT3, and HGF/Met signaling pathways [28–31]. Additionally, in the context of tumor microenvironment (TME) acidification, the export of lactate ions and H+ ions through hypoxia/HIF-1α-driven MCT4 enhances the acidity of the TME. This upregulates MMP-2 and MMP-9, leading to increased tissue-protein activity, EMT, and metastasis through augmented ROS formation [27, 32]. The acidic pH, resulting from lactic acid accumulation, also impedes the release of inflammatory cytokines essential for helper T cell polarization and differentiation of inflammatory dendritic cells [33, 34].

In this study, we identified the regulatory axis of *SLC2A3*/LA/TGF-β/SMAD in oral squamous cell carcinoma (OSCC). Initially, our western blot analysis revealed a one-way regulatory relationship from *SLC2A3* to TGF-β, echoing findings in colorectal cancer research by Li [35]. In contrast, other studies reported *SLC2A3* being regulated by TGF-β, which triggers JNK/ATF2 binding to its promoter region [36]. Nevertheless, our data predominantly show unidirectional regulation from *SLC2A3* to TGF-β, with the underlying mechanisms still unclear. The addition of exogenous lactic acid reactivated downstream TGF-β/SMAD signaling without significantly altering *SLC2A3* expression, suggesting lactic acid’s crucial role in connecting *SLC2A3* and TGF-β. Lactic acid is known to both directly and indirectly influence the TGF signaling pathway. For instance, lactic acid notably upregulates the transcriptional activator of TGF-β, thrombospondin-1 (THBS-1), via the ETS1/AP1 transcription factor, thus modulating TGF pathway activity [37]. Additionally, lactic acid can indirectly stimulate TGF-β activity by affecting the structure of intracellular TGF binding proteins (LTBP) during extracellular interactions [38]. Furthermore, lactic acid accumulation modifies the tumor microenvironment (TME), indirectly impacting TGF-β regulation. Studies have shown that a lactic acid-enriched TME enhances TGF-β expression in solid hypoxic cancers, which in turn regulates VEGF and MMPs expression, promoting angiogenesis [39, 40]. Moreover, the acidic pH in the TME, resulting from lactic acid accumulation, activates TGF-β1, further encouraging angiogenesis and invasiveness via SMAD signaling [41].

Still, The current study does have its constraints that must be recognized. Firstly, it is unknown what signalling LA regulates other than TGF-β. Secondly, other downstream signals besides SMADs in the TGF pathway activated by *SLC2A3* need to be further explored. Additionally, further research needs to be conducted to determine the effect of LA on more specific clinical outcomes in OSCC.

In conclusion, our study found that *SLC2A3* expression was up-regulated in OSCC and was associated with poor prognosis. *SLC2A3* promotes lactic acid production which activates the TGF-β signaling pathway, thus facilitating cell proliferation, migration and invasion in OSCC. Our study may provide new ideas for early prediction and treatment of OSCC.

## Supporting information

S1 Fig The IC50 of lactic acid. (TIF)
