# Supplementary figures and images for: SLC2A3 promotes tumor progression through lactic acid-promoted TGF-β signaling pathway in oral squamous cell carcinoma
Source: PLoS One. 2024 Apr 16;19(4):e0301724. doi: 10.1371/journal.pone.0301724 (PMC11020985; doi:10.1371/journal.pone.0301724)

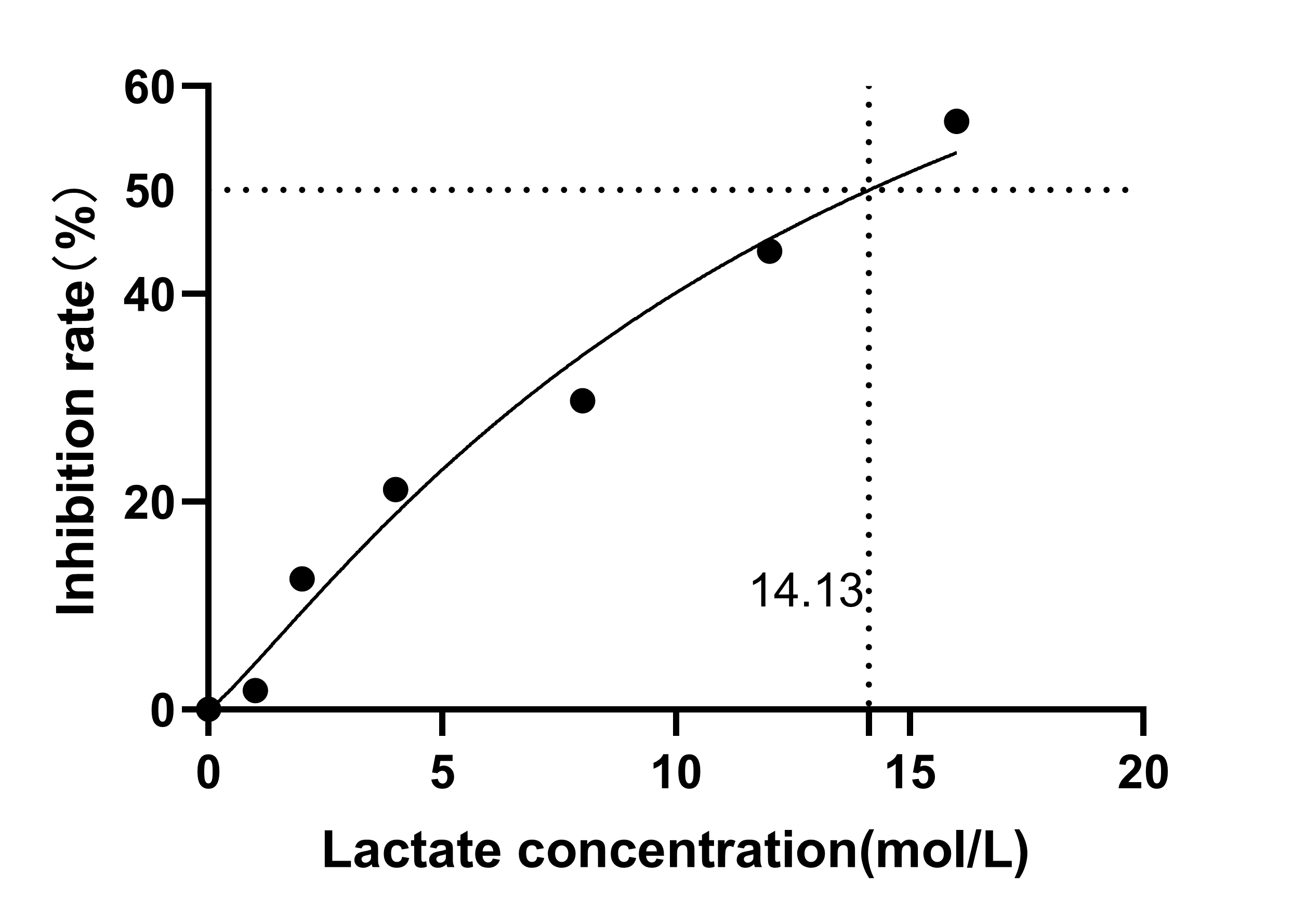

Supplement: S1 Fig — (TIF) [file pone.0301724.s001.tif]
